# The differential effects of metronomic gemcitabine and antiangiogenic treatment in patient-derived xenografts of pancreatic cancer: treatment effects on metabolism, vascular function, cell proliferation, and tumor growth

**DOI:** 10.1007/s10456-016-9503-z

**Published:** 2016-03-09

**Authors:** Donald T. Yapp, May Q. Wong, Alastair H. Kyle, Shannon M. Valdez, Jenny Tso, Andrew Yung, Piotr Kozlowski, David A. Owen, Andrzej K. Buczkowski, Stephen W. Chung, Charles H. Scudamore, Andrew I. Minchinton, Sylvia S. W. Ng

**Affiliations:** Department of Experimental Therapeutics, British Columbia Cancer Agency, 675 West 10th Avenue, Vancouver, BC V5Z 1L3 Canada; Faculty of Pharmaceutical Sciences, University of British Columbia, Vancouver, BC Canada; Integrative Oncology, British Columbia Cancer Agency, Vancouver, BC Canada; Magnetic Resonance Imaging Research Centre, University of British Columbia, Vancouver, BC Canada; Pathology and Laboratory Medicine, University of British Columbia, Vancouver, BC Canada; Department of Surgery, Faculty of Medicine, University of British Columbia, Vancouver, BC Canada; The Department of Radiation Oncology, Princess Margaret Cancer Centre, 5th Floor, 610 University Avenue, Toronto, ON M5G 2M9 Canada

**Keywords:** Metronomic chemotherapy, Anti-angiogenesis, Tumor physiology, Gemcitabine, Pancreatic cancer, Patient-derived xenografts, MRI, PET-CT

## Abstract

**Background:**

Metronomic chemotherapy has shown promising activity against solid tumors and is believed to act in an antiangiogenic manner. The current study describes and quantifies the therapeutic efficacy, and mode of activity, of metronomic gemcitabine and a dedicated antiangiogenic agent (DC101) in patient-derived xenografts of pancreatic cancer.

**Methods:**

Two primary human pancreatic cancer xenograft lines were dosed metronomically with gemcitabine or DC101 weekly. Changes in tumor growth, vascular function, and metabolism over time were measured with magnetic resonance imaging, positron emission tomography, and immunofluorescence microscopy to determine the anti-tumor effects of the respective treatments.

**Results:**

Tumors treated with metronomic gemcitabine were 10-fold smaller than those in the control and DC101 groups. Metronomic gemcitabine, but not DC101, reduced the tumors’ avidity for glucose, proliferation, and apoptosis. Metronomic gemcitabine-treated tumors had higher perfusion rates and uniformly distributed blood flow within the tumor, whereas perfusion rates in DC101-treated tumors were lower and confined to the periphery. DC101 treatment reduced the tumor’s vascular density, but did not change their function. In contrast, metronomic gemcitabine increased vessel density, improved tumor perfusion transiently, and decreased hypoxia.

**Conclusion:**

The aggregate data suggest that metronomic gemcitabine treatment affects both tumor vasculature and tumor cells continuously, and the overall effect is to significantly slow tumor growth. The observed increase in tumor perfusion induced by metronomic gemcitabine may be used as a therapeutic window for the administration of a second drug or radiation therapy. Non-invasive imaging could be used to detect early changes in tumor physiology before reductions in tumor volume were evident.

**Electronic supplementary material:**

The online version of this article (doi:10.1007/s10456-016-9503-z) contains supplementary material, which is available to authorized users.

## Background

The prognosis for pancreatic adenocarcinoma remains dire, with a 5-year survival rate of <5 % [1]. The clinical response to gemcitabine is significant, but its effect on overall survival is modest [2]. In addition, gemcitabine’s toxic side effects can be dose limiting and some pancreatic tumors are inherently resistant to the drug. At present, conventional chemotherapy for pancreatic cancer consists of maximum tolerated doses (MTD) of gemcitabine, in which the patient is given the highest possible drug dose that does not cause life-threatening side effects. The inherently toxic nature of MTD treatment requires drug-free breaks to allow the patient to recover from systemic drug toxicities before resuming the treatment. Unfortunately, the tumor often reestablishes itself during the drug-free breaks, and sometimes with acquired resistance to gemcitabine, that renders subsequent cycles of treatment ineffective. More effective strategies for treating and controlling pancreatic cancer are thus needed.

An alternate treatment regimen for pancreatic cancer under investigation is metronomic chemotherapy where low doses of a cytotoxic drug are administered frequently without prolonged drug-free breaks [8]. Drug doses lower than MTD, even if given more frequently and without rest breaks, are tolerated better by patients and cause fewer side effects. Interestingly, studies also indicate that drug resistant tumors can still respond to metronomic dosing of the same drug [3, 4], implying that direct cytotoxic kill of cancer cells is not the only mechanism of tumor control. Cytotoxic agents also target proliferating endothelial cells in the tumor [5], and it is not unreasonable that metronomic chemotherapy exerts non-specific effects on tumor vasculature that limits the supply of oxygen and nutrients and subsequently impedes tumor growth [6–8]. Moreover, the lack of treatment breaks in metronomic chemotherapy also means that the continuous presence of drug prevents damaged vasculature from recovering [3]. The effects of metronomic chemotherapy on tumor vasculature are largely unknown except for a handful of studies where increased vessel perfusion was observed [9–11].

We now report here the different effects that metronomic gemcitabine and DC101 treatment have on two patient derived pancreatic tumor lines grown orthotopically in mice. Our data show that metronomic therapy with gemcitabine not only improves the tumor’s vascular function transiently, but also significantly retards cell proliferation and metabolism in primary human pancreatic cancer xenografts. In contrast, DC101, a monoclonal antibody that targets mouse vascular endothelial growth factor receptor-2 (VEGFR-2) decreases vascular density as expected but had no effect on tumor growth in the same models. The molecular and in situ physiological data reported here provide more insight on the activity of metronomic gemcitabine in primary pancreatic tumors. The information garnered suggests that the activity of Met-Gem treatment is not only cytotoxic, but also affects tumor vasculature effects as well. The data further suggest a role for non-invasive imaging technologies in monitoring changes in the tumor microenvironment, which could be used to guide the development of more effective therapies and dosing parameters specifically for pancreatic cancer.

## Methods

### Orthotopic primary pancreatic cancer xenografts

Fresh pancreatic ductal adenocarcinoma (PDAC) tissues were obtained from consented patients undergoing Whipple resection at Vancouver General Hospital in 2006–2008. Resected, non-diagnostic specimens were collected as previously described [9, 12]. Briefly, viable tumor tissues were implanted subcutaneously into male C.B-17 SCID mice (Taconic, Germantown, NY, USA). When tumor volumes reached 600–800 mm^3^, the subcutaneous tumors were excised, cut into small pieces, and surgically implanted on the pancreas of additional mice. Two primary pancreatic ductal adenocarcinoma xenograft lines, PaCa8 and PaCa13, established from two patients were used in this study. All animal studies were done in accordance with the guidelines from the Canadian Council for Animal Care and approved by the University of British Columbia’s animal care committee.

### Treatments and in vivo imaging time points

**Scheme 1:**
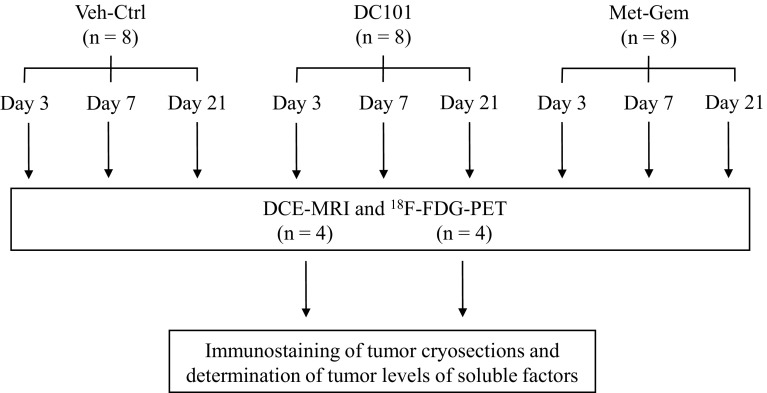
Experimental design. PaCa8 or PaCa13 xenografts were implanted into 24 mice each. When tumors were 200–400 mm^3^, mice were randomized into one of three treatment groups: vehicle (Veh-Ctrl), anti-VEGFR-2 antibody (DC101), or metronomic gemcitabine (Met-Gem). On days 3, 7 and 21 after treatment started, animals were imaged with DCE-MRI or FDG-PET. Animals were euthanized immediately after imaging, and the tumors harvested for further analysis

Gemcitabine hydrochloride (Gemzar^®^; Eli Lilly Canada Inc., Toronto, ON, Canada) was obtained from the British Columbia Cancer Agency (BCCA) pharmacy. Mouse monoclonal VEGFR-2 antibody, DC101, was provided by Imclone Systems (New York, NY, USA) via a materials transfer agreement. When the orthotopic primary xenografts reached a palpable size of 200–400 mm^3^, tumor-bearing mice were randomly assigned to three groups (*n* = 8/group) and treated with vehicle control (Veh-ctrl; 0.9 % saline, i.p.), DC101 (800 μg, q3d, i.p.), or metronomic gemcitabine (Met-Gem; 30 mg/kg, q3d, i.p.). Gemcitabine doses were chosen to emulate typical serum concentrations in the clinic as reported previously [9], and DC101 doses were based on previous studies that showed efficacy [13, 14]. On days 3, 7, and 21 following initiation of treatment, mice from each group were scanned with dynamic contrast-enhanced magnetic resonance imaging (DCE-MRI) or ^18^F-fluorodeoxyglucose positron emission tomography (FDG-PET). Once imaging procedures were complete, mice were euthanized and tumors harvested for immunostaining or molecular interrogation (See Scheme 1 for experimental flow).

### ^18^F-fluorodeoxyglucose positron emission tomography (FDG-PET)

At each time point, tumor-bearing mice were injected with FDG (^18^F-radiolabeled deoxyglucose; 100 ± 5 μCi; BCCA) via the lateral tail vein. One hour after injection, the mice were anesthetized with isoflurane and placed on the scanning platform. The Inveon multi-modality small animal PET-CT scanner (Siemens, Knoxville, TN, USA) was used for imaging studies. PET data were collected for 15 min in list mode and subsequently histogrammed into a single frame. Images were reconstructed in three dimensions using OSEM-MAP3D algorithms following CT-based attenuation scans to correct for the animal’s body mass. Three-dimensional regions of interest were placed on parts of the tumor that were actively taking up FDG in the reconstructed animal images to quantify FDG activity present per volume of tumor tissue. FDG-PET imaging is based on detecting the uptake of FDG by cancer cells. The avidity of the cells for FDG, and by extension glucose, is a gauge of the tumor’s proliferative ability and viability [15]. Generally, the more FDG is taken up by a tumor, the more viable and proliferative the tumor is. In the clinic, FDG-PET images are used to stage tumors and diagnose metastatic disease [15, 16]. CT images, based on tissue densities and injected contrast agents, provide information on the physical volume of the tumor. The FDG signal is used to corroborate the CT image of the tumor. Changes in the FDG signal occur more rapidly than changes in tumor volume, so this information can also be used to infer early response to a treatment—i.e., if the FDG signal is reduced by a treatment (compared to a baseline scan before treatment), it indicates that the cells are less viable and proliferative. We have used PET data reported here in a similar fashion to provide additional information on the effects of metronomic gemcitabine and DC101 on the viability and proliferative ability of the tumor before and after treatment.

### Immunofluorescence staining, microscopy, and image analysis

Following DCE-MRI and FDG-PET, all tumor-bearing mice were injected with the hypoxia marker EF5 (30 mg/kg, i.v.; Dr. Cameron Koch, University of Pennsylvania, PA, USA) and the perfusion marker Hoechst 33342 (40 mg/kg, i.v.; Sigma-Aldrich, Oakville, ON, Canada) at 3 h and 5 min, respectively, before euthanasia and immediate tumor excision. Perpendicular tumor diameters were measured with a caliper, and tumor volumes were calculated (π/6 × a × b^2^, where ‘a’ is the longest dimension of the tumor, and ‘b’ is the width). Tumors were preserved in OCT (Sakura Finetek, Torrance, CA, USA) and snap-frozen in liquid nitrogen vapor. A known value of 10-μm cryosections were cut from each tumor, air-dried, and imaged first for native Hoechst 33362 fluorescence as an indicator of tumor tissue perfusion [17]. Subsequently, the cryosections were fixed in 50 % (v/v) acetone/methanol and blocked in a buffer containing 1 % BSA, 1 % goat serum, 1 % donkey serum, and 0.1 % Tween-20. Endothelial cells were stained with a CD31/PECAM-1 primary antibody (1:1000; BD PharMingen, San Diego, CA, USA) followed by an Alexa 647-conjugated secondary antibody (1:20,000; Invitrogen, Burlington, ON, Canada). Reduced EF5 adducts in viable hypoxic cells were stained using a Cy3-conjugated monoclonal antibody to ELK3-51 (1:400; Dr. C. Koch). To visualize proliferating cells, cryosections were stained with a Ki67 (1:100; Invitrogen) primary antibody followed by an Alexa 488 conjugated secondary antibody (1:200; Invitrogen). The in situ Cell Death Detection Kit (Roche Indianapolis, IN, USA), which involves TdT-mediated dUTP nick end labeling (TUNEL) reaction, was used to evaluate cell apoptosis. Finally, cryosections were counterstained with Hoechst 33362 (10 μg/mL) and mounted. Fluorescent images of whole tumor sections were captured (×10 objective) using a microscope system (Leica Microsystems Inc., Richmond Hill, ON, Canada) equipped with a cooled 350FX monochrome CCD camera, an automated scanning stage (DM6000B), and Surveyor software (Objective Imaging Kansasville, WI, USA). All parameters stained on the same cryosection were imaged separately and then overlaid and aligned. With NIH ImageJ (http://rsb.info.nih.gov/ij/) software and user-supplied algorithms, images were cropped to tumor tissue boundaries with necrosis and artifacts removed before quantification. The extent of hypoxia, proliferation, and apoptosis was represented by the percentage of EF5+, Ki67+, and TUNEL+ pixels, respectively, of the total number of viable tumor tissue pixels. The percentage of perfused vessels was reported as the percentage of CD31 + Hoechst + pixels divided by the total number of CD31 + pixels. To measure microvessel density, all image pixels were sorted to determine their distance to the nearest CD31 + pixel, and the average of distances to the nearest vessel (i.e., CD31 + object) was calculated. A short average distance to the nearest vessel indicates high microvessel density [9, 18]. The degree of necrosis present in the tumors was calculated as a percentage of necrotic pixels divided by total tumor tissue pixels; necrotic areas were identified based on side-by-side comparisons with H&E images of the section.

### Dynamic contrast-enhanced magnetic resonance imaging (DCE-MRI)

A 7.0 Tesla MR scanner (Bruker, Ettlingen, Germany), with a quadrature birdcage coil for transmission and a 1.7 × 1.4 cm rectangular surface coil for reception, was used for DCE-MRI studies. At each time point, tumor-bearing mice were anesthetized with isoflurane (Baxter Corporation, Mississauga, ON, Canada) and given an i.v. bolus injection of 0.3 mmol/kg of the MR contrast agent gadodiamide (Omniscan™; Nycomed, Oslo, Norway). 3D-FLASH was used to acquire the data for estimating the concentration of the contrast agent (FOV = 3.84 × 21.6 × 2.4 cm; voxel size = 0.3 × 0.3 × 1 mm): three scans with different flip angles were used to calculate in vivo flip angle maps [19] in order to correct the T_1_ estimates (α_nom_ = 145°, 180°, 215°; TE/TR = 3.5/460 ms, 2 × zero-filling for 0.3 × 0.3 × 1 mm voxel); a three-scan variable flip angle method was used to calculate native T_1_ in the tumor [20] (α_nom_ = 10°, 20°, 50°; TE/TR = 2.7/144 ms); and a rapid T_1_-weighted scan series (TE/TR = 2.7/9 ms, α_nom_ = 25°, 15.6 s per scan) was performed before and after bolus injection of the contrast agent (20 pre-contrast scans, 150 post-contrast scans) to observe the initial uptake and subsequent washout in the tumor. Concentration was derived assuming linearity between contrast concentration and T_1_ according to the equations described by Schabel and Parker [21]. The gadodiamide concentration–time curve for each pixel was fitted to the extended Kety model [22, 23] which describes the pharmacokinetics of the contrast agent using three parameters: *K*^trans^—volume transfer constant between vascular space and extravascular extracellular space; *v*_e_—fractional volume of extravascular extracellular space; and *v*_p_—fractional volume of vascular space. The arterial input function was derived from a population average in the same tumor model as previously described [24]. *K*^trans^, which is often a mixed measure of blood flow and vascular permeability [25], was determined using in-house software and expressed as median values of *K*^trans^ in viable tumor tissues. To assess *K*^trans^ in the tumor periphery versus tumor core, three-dimensional *K*^trans^ voxel maps were processed by binary erosion [26], which segmented the *K*^trans^ voxel distribution into three-dimensional concentric shells that were each a single voxel thick. Using this segmentation of voxels, *K*^trans^ was averaged from the outer one-third and inner two-thirds of tumor voxels to represent the tumor periphery and the tumor core, respectively.

Magnetic resonance imaging is used extensively in the clinic for high-resolution anatomical imaging [15, 27]. MR anatomical images are based on detecting water molecules under a magnetic field and the differences between water molecules in different environments. In some cases, MRI is also used to examine perfusion or blood flow in a tumor using contrast agents and specialized imaging sequences [27]. We have taken a similar approach in our study, and imaged/quantified the effects of an injected contrast agent as it appears and exits the tumor via the blood vessels. The data are then processed to provide us with an estimate of perfusion rates in the tissue before and after treatment. In this way, we are able to associate the overall vascular function of the tumor following Met-Gem and DC101 treatment.

### Tumor levels of pro- and antiangiogenic factors

Harvested tumors from the three treatment groups at each time point were snap-frozen in liquid nitrogen and homogenized in a lysis buffer (50 mM HEPES, 10 % glycerol, 1 % Triton X-100, 150 mM NaCl, 1 mM EDTA, 1.5 mM MgCl_2_, 100 mM NaF, 10 mM Na_4_P_2_O_7_, 100 μg/ml PMSF, 5 μg/ml leupeptin, and 5 μg/ml aprotinin) using a Polytron PT10-35 homogenizer (Kinematica AG, Lucerne, Switzerland) and stored at −80 °C. Total protein concentration in each tumor lysate was determined using a Micro BCA proteins assay kit (Pierce. Rockford, IL, USA). The protein levels of human vascular endothelial growth factor (hVEGF), mouse vascular endothelial growth factor (mVEGF), mouse platelet-derived growth factor-BB (mPDGF-BB), mouse stromal cell-derived factor-1α (mSDF-1α), human placental growth factor (hPlGF), and human thrombospondin-1 (hTSP-1) were determined using ELISA kits (R&D Systems. Minneapolis, MN. USA).

### Statistics

All results were presented as mean ± SE. Comparisons were made with one-way analysis of variance followed by the Newman–Keuls test, with *P* < 0.05 as the criterion for statistical significance.

## Results

### Metronomic gemcitabine is more effective than DC101 in controlling tumor growth and decreasing tumor metabolism

**Fig. 1:**
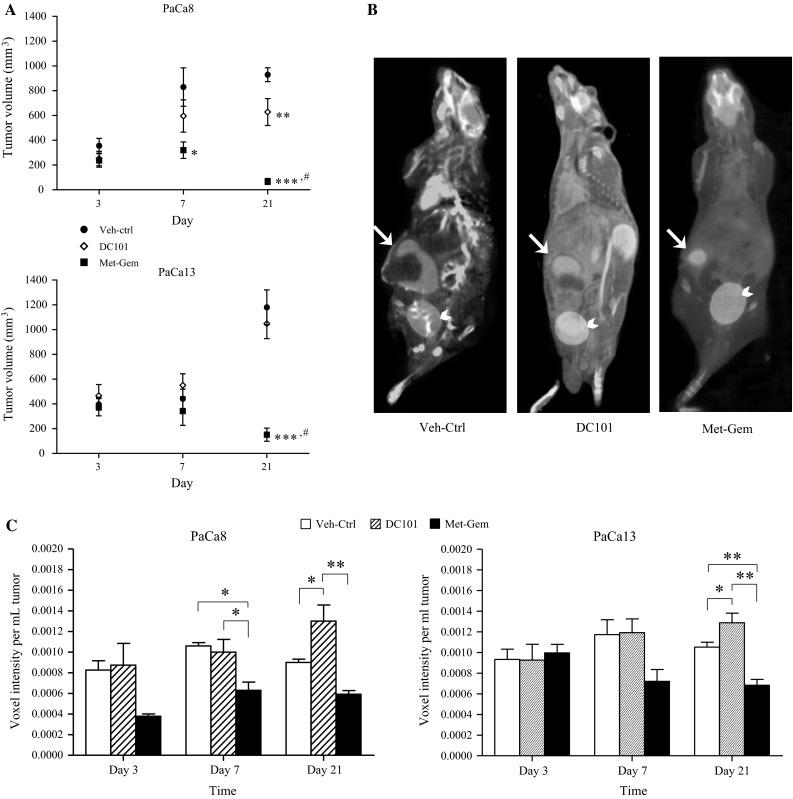
Metronomic gemcitabine reduces the growth and metabolic activity of pancreatic tumors. **a** Effects of treatments on the volume of PaCa8 and PaCa13 tumors on days 3, 7, and 21 after initiation of treatment. *Symbols*, mean of 6–11 tumors; *bars*, SE. *P < 0.05, **P < 0.01, or ***P < 0.005 versus vehicle control of corresponding day; ^#^P < 0.01, versus DC101 of corresponding day. **b** Representative FDG-PET-CT images of SCID mice bearing PaCa13 tumors in the pancreas on day 7 after initiation of treatment. Tumor, *white arrow*; bladder, *white arrow heads*. **c** Effects of treatments on FDG uptake in PaCa8 and PaCa13 tumors at the three time points. *Columns*, mean of 2–6 tumors; *bars*, SE. *P < 0.05; **P < 0.01

Metronomic gemcitabine (Met-Gem) treatment significantly reduced the volume of PaCa8 and PaCa13 tumors compared to the time-matched vehicle controls (93 and 87 %; *P* < 0.01, respectively, Fig. 1a) by day 21. In contrast, no significant changes in tumor size were observed with DC101 treatment, except in PaCa8 tumors on day 21 when the volumes were reduced by 32 % (*P* < 0.01). Throughout the course of treatment with DC101 or Met-Gem, no significant changes in weight or adverse effects were observed in the mice indicating that the treatments were well tolerated.

Representative FDG-PET-CT images of PaCa13 tumor-bearing mice on day 7 post-treatment initiation show that tumor edges are well delineated by FDG uptake (Fig. 1b); significant differences in tumor size among the three groups (Veh-ctrl, DC101, and Met-Gem) are already apparent at this time point. Mice treated with Met-Gem had the smallest tumors of the three groups, and few necrotic regions were present (areas with no FDG uptake) within the tumor. In contrast, PET images of the Veh-ctrl and DC101 tumors show the presence of a central necrotic core with FDG uptake found predominantly in the proliferating tumor edge.

The metabolic activity of tumors in the treatment groups was also evaluated at all time points by measuring the uptake of FDG per unit volume of tumor tissue. The average voxel intensity measurements, normalized to the tumor volume, in each group of tumors are shown in Fig. 1c. The voxel intensity is proportional to the amount of FDG taken up by the cells in the tissue and is an indication of the tissue’s metabolic activity. The overall FDG-PET data show that Met-Gem treatment decreased tumor metabolism, whereas DC101 treatment actually increased it. Compared to Veh-ctrl tumors, Met-Gem treatment significantly reduced metabolic activity by ~40 % (*P* < 0.05) in PaCa8 and PaCa13 tumors on day 7. With the exception of PaCa13 tumors on day 3, a trend toward decreased metabolic activity for both pancreatic tumor xenograft lines was seen following treatment with Met-Gem. Treatment with DC101, however, did not change the metabolic activity of the tumors (compared with controls) on days 3 and 7; however, on day 21, the metabolic activity increased significantly (*P* < 0.05) by 44 and 23 % in PaCa8 and PaCa13 tumors, respectively (Fig. 1c).

**Fig. 2:**
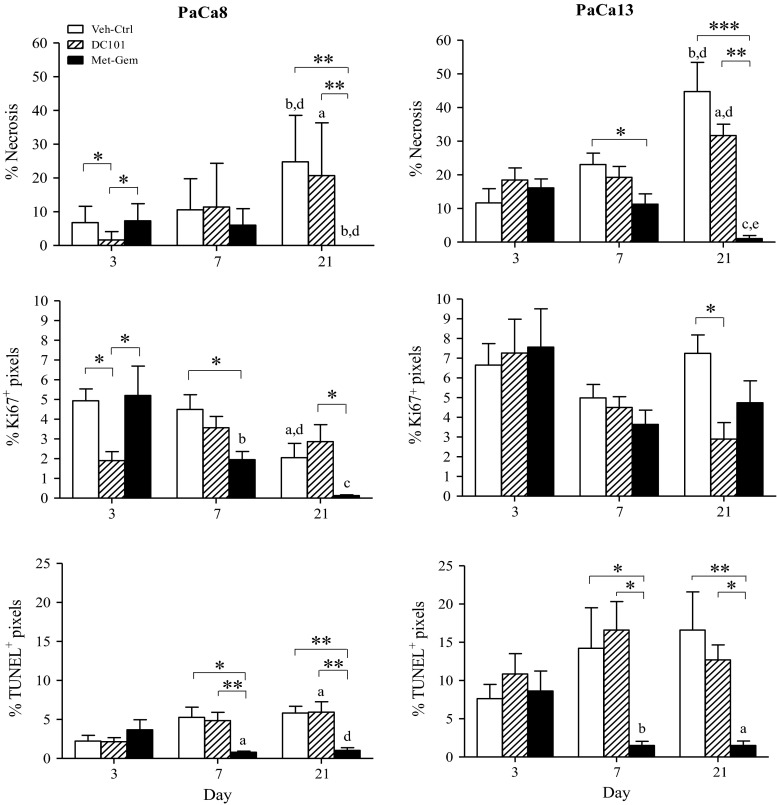
Metronomic gemcitabine-treated tumors are less necrotic and have lower levels of proliferation and apoptosis compared to DC101-treated tumors. Immunofluorescence staining of tumor sections was quantified for necrosis, proliferation (Ki67^+^ staining), and apoptosis (TUNEL^+^ staining). *Columns*, mean of 6–8 tumors; *bars*, SE. *P < 0.05; **P < 0.01; ***P < 0.005. ^a^ *P* < 0.05, ^b^ *P* < 0.01, or ^c^ *P* < 0.001, versus day 3 of corresponding treatment group. ^d^ *P* < 0.05 or ^e^ *P* < 0.01, versus day 7 of corresponding treatment group

The percent necrotic tissue present in the tumors was calculated, and the results show that by day 21 of treatment, the Met-Gem groups (PaCa8 and PaCa13 tumors) were significantly less necrotic than the Veh-ctrl group (*P* < 0.01; Fig. 2). In contrast, DC101 treatment only reduced necrosis in PaCa8 tumors on day 3, and the extent of necrosis at all other time points was similar between DC101-treated and Veh-ctrl tumors. Excised tumors were also analyzed for cell proliferation (Ki67) and death (TUNEL) with immunofluorescence staining. Quantification of Ki67 and TUNEL staining (Fig. 2) revealed that levels of Ki67^+^ and TUNEL staining in tumors from the Met-Gem treatment groups decreased over time in PaCa8 and Pac13 tumors compared to Veh-ctrl. The extent of Ki67 and TUNEL staining in DC101-treated tumors were generally the same as Veh-ctrl tumors except in PaCa8 tumors on day 3 when levels of Ki67 were significantly lowered.

### Tumors treated with metronomic gemcitabine have a higher density of blood vessels that are more functional

**Fig. 3:**
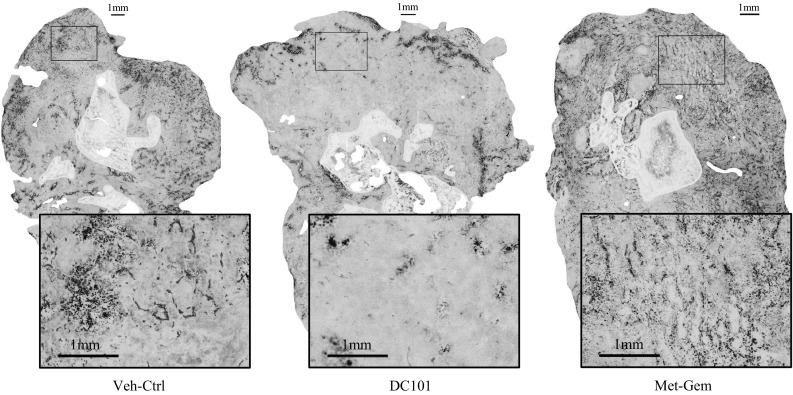
Metronomic gemcitabine-treated tumors are better perfused, have higher intratumoral microvessel density, and lower levels of hypoxia than DC101-treated tumors. Representative composite images of PaCa13 tumor sections on day 7 of treatment with vehicle (Veh-Ctrl; 0.9 % saline), DC101 (800 μg, q3d), or metronomic gemcitabine (Met-Gem; 30 mg/kg, q3d). The presence of Hoechst 33362 (*blue*) indicates tissue perfusion. Intratumoral microvessels and hypoxia are detected by CD31 (*red*) and EF5 (*green*) immunofluorescence staining, respectively. Necrotic regions (*pale green*) are outlined in *yellow*

**Fig. 4:**
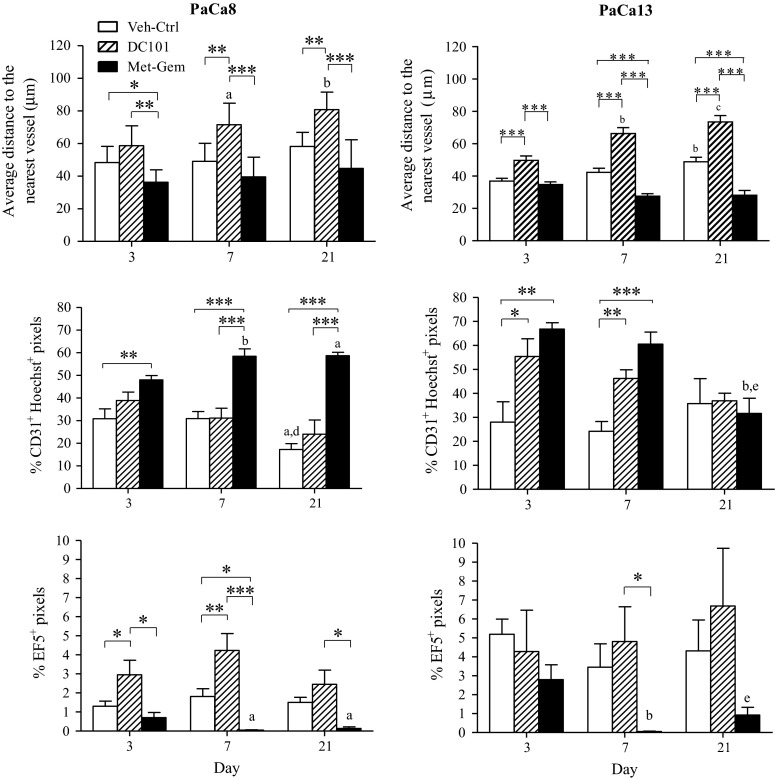
Intratumoral microvessel density, tissue perfusion, and tumor hypoxia in tumors treated with saline, DC101, and Met-Gem. Immunofluorescence staining of tumor sections was quantified for intratumoral microvessel density (average distance of tissue pixel to the nearest vessel), percent perfused vessels (CD31^+^Hoechst^+^ staining), and percent hypoxic areas (EF5^+^ staining). *Columns*, mean of 2–9 tumors; *bars*, SE. *P < 0.05; **P < 0.01; ***P < 0.005. ^a^ *P* < 0.05, ^b^ *P* < 0.01, or ^c^ *P* < 0.005, versus day 3 of corresponding treatment group. ^d^ *P* < 0.05 or ^e^ *P* < 0.01, versus day 7 of corresponding treatment group

Representative composite images of PaCa13 tumor sections from each treatment group (day 7) are shown in Fig. 3 (See Additional File 1 for PaCa8 images). The images clearly show that fewer necrotic regions (areas outlined in yellow) are present in tumors treated with Met-Gem as indicated by the previously (Fig. 2) and that the vessels (red) are more evenly distributed throughout the tumor than in Veh-ctrl- and DC101-treated tumors. Selected vascular parameters for the stained images were quantified and are summarized in Fig. 3. The density of blood vessels in tumors from each treatment group was determined by averaging the distance between tissue pixels to the nearest CD31^+^ pixel, where shorter distances indicate higher vessel density. With the exception of PaCa8 tumor (day 3), all DC101-treated tumors had significantly (*P* < 0.01) lower vessel density than Veh-ctrls and the vessel density decreased even more with time. Only PaCa13 tumors treated with Met-Gem had a significantly higher density of blood vessels (*P* < 0.005) on days 7 and 21 when compared to Veh-ctrl tumors. Overall, vascular density in the metronomic gemcitabine-treated tumors were significantly (*P* < 0.005) greater than the DC101-treated tumors (Figs. 3, 4).

The levels of double positive CD31 and Hoechst pixels in the tumor were quantified to evaluate the number of blood vessels that contained Hoechst as an indicator of vessel function at the time of injection. This analysis revealed that, compared to the Veh-ctrl tumors, significantly more blood vessels in Met-Gem-treated tumors were carrying the dye except for PaCa13 on day 21 (Fig. 4; *P* < 0.05). By day 7 of Met-Gem treatment, the number of vessels positive for Hoechst staining for PaCa8 and PaCa13 tumors had increased by ~twofold compared to Veh-ctrl tumors. On days 3 and 7 after initiation of treatment, DC101-treated PaCa13 tumors also contained significantly (*P* < 0.05) more Hoechst than their Veh-ctrl counterparts. This was not the case for DC101-treated PaCa8 tumors, however, and the number of Hoechst positive blood vessels was similar to controls.

The levels of hypoxia in PaCa8 and PaCa13 were also evaluated using the exogenous hypoxia marker, EF5 [28, 29]. The drastic decrease in hypoxia is seen visually by the lack of EF5 adducts present in the cells (Fig. 3, green stain) of Met-Gem-treated tumors. Quantification of the EF5 positive pixels in the tumors (Fig. 4) shows that the number of hypoxic cells present is reduced, and significantly so by day 7 (PaCa8 and PaCa13) and 21 (PaCa8). Tumor hypoxia in DC101-treated tumors was similar to that in Veh-ctrl tumors, and in fact a significant (*P* < 0.05) increase in hypoxia was seen in PaCa8 tumors on day 7. Overall, quantification of hypoxic cells in the sections indicates that Met-Gem treatment decreased hypoxia while DC101 treatment had no effect or increased hypoxia in the tumors (Fig. 4).

### Metronomic gemcitabine-treated tumors are better perfused than DC101-treated tumors

**Fig. 5:**
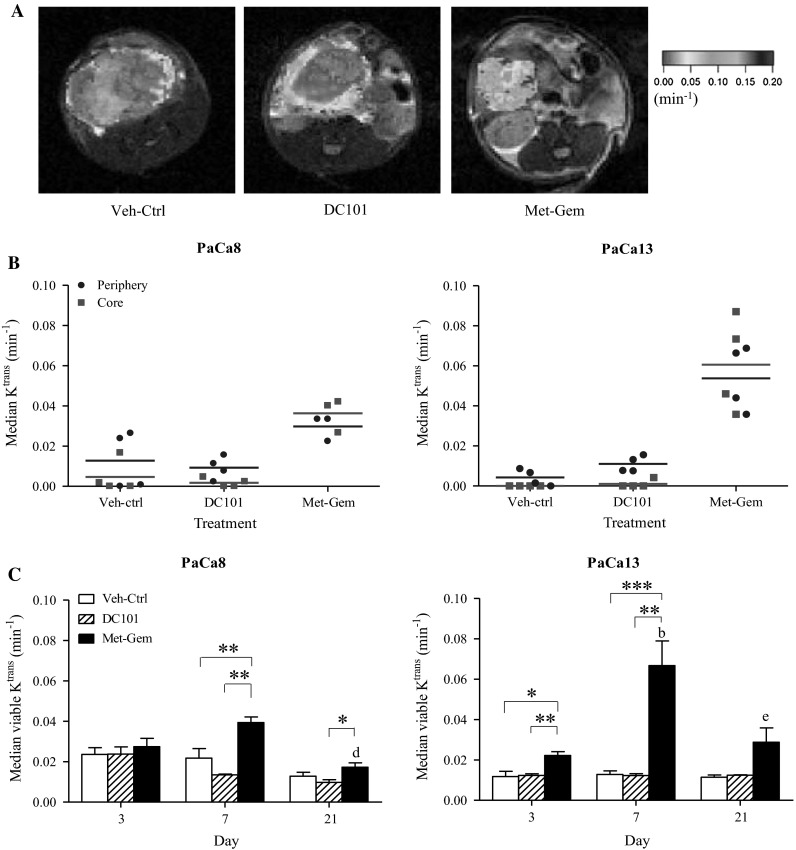
Metronomic gemcitabine improves tumor perfusion in primary pancreatic tumors. **a** Representative *K* ^trans^ maps of PaCa13 tumors on day 7 of treatment are superimposed on the corresponding axial anatomical MR images. Higher *K* ^trans^ values indicate better perfusion. **b** Comparison of median *K* ^trans^ values between the outer one-third (periphery) and inner two-thirds (core) of PaCa8 and PaCa13 tumors on day 7 after initiation of treatment. *Lines*, mean of 3–4 tumors. **c** Effects of treatments on median *K* ^trans^ values at the three time points. *Columns*, mean of 3–4 tumors; *bars*, SE. *P < 0.05; **P < 0.01; ***P < 0.005. ^b^ *P* < 0.01, versus day 3 of corresponding treatment group; ^d^ *P* < 0.05 or ^e^ *P* < 0.01, versus day 7 of corresponding treatment group

Representative *K*^trans^ maps of PaCa13 tumors, 7 days after initiation of treatment, are shown in Fig. 5a. The presence of the contrast agent (Gd-DTPA) in the tumor is indicated by colored areas where red and blue areas indicate low and high concentrations of Gd-DTPA, respectively. The presence of Gd-DTPA indicates areas of tissue that are actively perfused at the time of the scan, and its concentration a measure of relative perfusion rates. The highest levels of perfusion in tumors from the Veh-ctrl and DC101 groups were found in the periphery (green–blue areas), while the center of these tumors either had no (gray unmapped areas) or low perfusion (red areas). In tumors treated with Met-Gem, however, not only were higher levels of the contrast agent (blue-green) found, but the contrast agent was more uniformly distributed throughout the tumor. The median *K*^trans^ values of the outer, one-third periphery of the tumor and those of the inner, two-third core of the tumor, 7 days after initiation of treatment median are shown in Fig. 5b, and quantitatively confirm the difference in perfusion rates observed in the color maps. Median *K*^trans^ levels in the periphery of Veh-ctrl- and DC101-treated tumors (PaCa8 and PaCa13) are higher than in the core, where they are almost negligible. In contrast, median *K*^trans^ values in Met-Gem-treated tumors were higher in the tumor core than in their periphery. In general too, the overall *K*^trans^ values in Met-Gem-treated tumors were higher than those found in Veh-ctrl and DC101-treated tumors indicating that Met-Gem treatment improves the overall perfusion in the tumor.

The average of median *K*^trans^ values derived from the entire MRI-pixel map of the tumor on days 3, 7, and 21 is shown in Fig. 5c. Median *K*^trans^ values for DC101-treated tumors and Veh-ctrl tumors were similar at all time points in both PaCa8 and PaCa13. In contrast, tumors treated with Met-Gem for 7 days had significantly (*P* < 0.01) higher *K*^trans^ values than those treated with Veh-ctrl or DC101 (~twofold and ~fivefold, respectively, for PaCa8 and PaCa13 tumors compared to their corresponding controls). Interestingly, after 21 days of Met-Gem treatment, the median *K*^trans^ values dropped significantly (*P* < 0.05) compared to day 7 values. The data graphed in Fig. 5c show that Met-Gem treatment improves tumor perfusion transiently, whereas DC101 had little or no effect on the tumors.

### Metronomic gemcitabine decreases the levels of pro- and anti-angiogenic factors

**Fig. 6:**
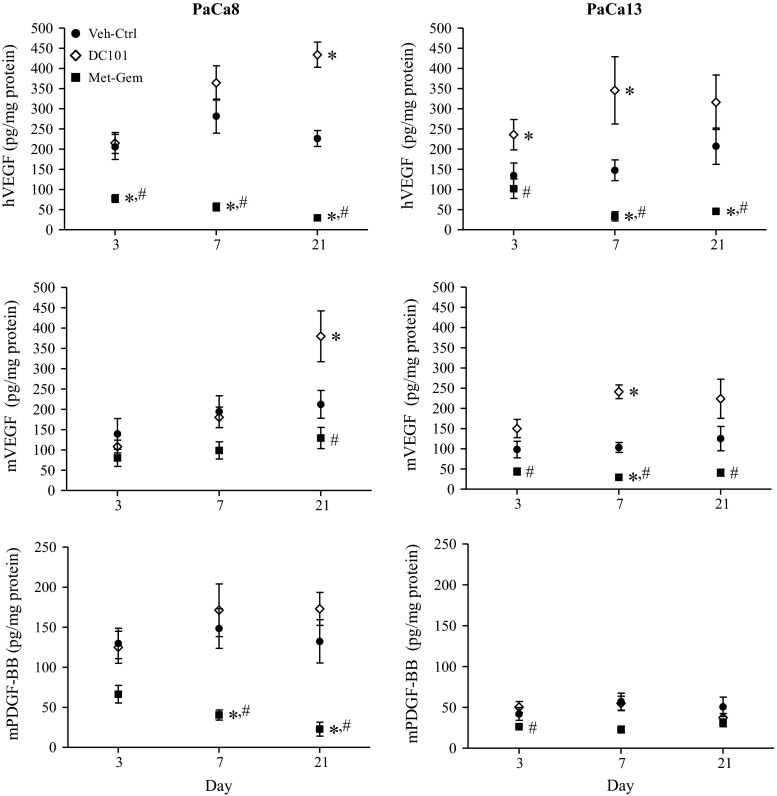
The effects of Met-Gem and DC101 on pro- and antiangiogenic factors. Metronomic gemcitabine decreases the levels of human and mouse vascular endothelial growth factor (hVEGF and mVEGF, respectively), and mouse platelet-derived growth factor (mPDGF-BB) in tumors. Tumor-bearing SCID mice were treated with the vehicle (Veh-Ctrl; 0.9 % saline), DC101 (800 μg, q3d), or metronomic gemcitabine (Met-Gem; 30 mg/kg, q3d) and were euthanized on days 3, 7, and 21 following initiation of treatment. Effects of treatments on hVEGF, mVEGF, and mPDGF-BB were measured with ELISAs. *Symbols*, means of 3–9 tumors; *bars*, SE. *P < 0.05, versus the vehicle control group at the same time point. ^#^ *P* < 0.05, versus the DC101-treated group at the same time point

The levels of pro- and antiangiogenic factors as detected by ELISA in the tumor samples are shown in Fig. 6. In both PaCa8 and PaCa13 tumors on day 7 and 21 following initiation of treatment, Met-Gem significantly (*P* < 0.05) decreased the levels of the proangiogenic factors hVEGF and mVEGF. On the other hand, DC101 increased the levels of hVEGF and mVEGF, but this could be attributed to the fact that DC101 is an antibody against VEGFR-2, which would increase levels of free VEGF. The other proangiogenic factor, mPDGF-BB, was also significantly (*P* < 0.05) decreased over time with Met-Gem treatment in PaCa8 tumors. Met-Gem and DC101 did not significantly alter the levels of hP*l*G, mSDF-α, and hTSP-1 (see Additional File 2).

## Discussion

The aim of this study was to better understand the anti-tumor effects of metronomic gemcitabine (Met-Gem) in the context of pancreatic adenocarcinoma. Our data clearly show that Met-Gem is very effective at controlling pancreatic tumor growth, with the average volume of Met-Gem-treated tumors almost 10 times smaller than that of Veh-ctrl tumors (Fig. 1a). The avidity of Met-Gem-treated tumors for FDG, a radiolabelled glucose analog, was measured with PET to evaluate the tumors’ glucose requirements and metabolic activity as a surrogate marker for in situ cell viability and proliferation. The PET data show that FDG is distributed homogenously in Met-Gem-treated tumors, which have few necrotic areas, but that the tumors were significantly less avid for FDG compared to Veh-ctrl tumors. Interestingly, FDG uptake in tumors treated with Met-Gem was lower as soon as day 3, whereas tumor volume data gave no indication of any response to metronomic treatment at this early time point (Fig. 1a, c). The levels of Ki67 and TUNEL staining in frozen sections from the Met-Gem-treated tumors (Fig. 2) were also lower than those in the Veh-ctrl tumors, consistent with the FDG data (Fig. 1). Met-Gem treatment, therefore, appears to exert a cytostatic effect on the tumor wherein the cancer cells are still alive, but not actively proliferating.

Metronomic dosing with other drugs has been reported to decrease tumor perfusion [3, 5, 30, 31], thus treatment induced changes and function in the tumors’ vasculature were examined using immunohistochemistry and DCE-MRI. The density and functionality of the tumor vasculature in our study were quantified, and the data (Fig. 4) indicate that Met-Gem treatment increases vascular density and improved function compared to Veh-ctrl tumors. Hypoxic cells were virtually absent in Met-Gem-treated tumors (Figs. 3, 4) and this is likely due to a more functional vascular system which delivers more oxygen and lower metabolic rates. Similar findings have also been reported in a breast cancer model treated with orally administered, low-dose gemcitabine [11].

The *K*^trans^ data (Fig. 5) indicate blood flow is homogenous throughout the tissue following Met-Gem treatment. Moreover, the average *K*^trans^ values increase by day 7 compared to Veh-ctrl tumors even though the average tumor volume in all groups are similar. The *K*^trans^ values, however, decrease by day 21, suggesting that the changes in tumor perfusion due to Met-Gem is dynamic and that the initial increase in perfusion observed is transient. Other studies report that treatment with Met-Gem decreases tumor perfusion [30], but this may be a reflection of when the scans were carried out with respect to the treatment as few longitudinal studies such as ours have been carried out.

Since Met-Gem treatment had such dramatic effects on the density and function of tumor vessels, we treated the same tumor models with DC101 to examine the effects of a dedicated antiangiogenic agent on tumor growth, metabolism, and vascular function. DC101, the murine analog of ramucirumab [32], was used because the vasculature that develops in the orthotopic tumors is of murine origin. DC101 therefore only targets actively growing tumor vasculature and has no effect on the human cancer cells. Our results indicate that DC101 treatment is much less effective than Met-Gem. Treatment with DC101 only reduced the volume of PaCa13 tumors by about 3 times after 21 days compared to Veh-ctrl tumors. The uptake and distribution of FDG in Veh-ctrl- and DC101-treated tumors were also similar—uptake was concentrated at the periphery, and the tumors had large central necrotic areas with no uptake. Moreover, the avidity of the DC101-treated and Veh-ctrl tumors for FDG was higher than that in Met-Gem-treated tumors indicating that the DC101-treated tumors, albeit highly necrotic, were still actively proliferating at the periphery, a pattern typical of rapidly growing tumors (Fig. 1). Similarly, levels of cell proliferation and apoptosis (Ki67 and TUNEL, respectively; Fig. 2) in DC101-treated tumors were similar to those present in Veh-ctrl tumors. Our data further show that DC101 was toxic to the vasculature (Fig. 4), but the consequences were not detrimental to tumor growth at least in these models and time points. As expected, DC101 reduced vascular density in the primary pancreatic tumor xenografts in our study as reported previously [13]; the same group also reported that DC101 reduced tumor volume [13], whereas no significant effects on tumor volume were seen in our study. Unfortunately, it is not possible to draw any definite conclusions between the two studies as our group used primary orthotopic human tumor xenografts [9], which bears the histology of the original resected patient tumor, whereas the other group used tumors derived from cell lines which tend to contain homogeneous sheets of cancer cells and a well distributed vascular system.

DC101 treatment did not change in situ tumor perfusion as evaluated with DCE-MRI over time either, and in fact, the median *K*^trans^ values in tumors treated with DC101 were similar to those in Veh-ctrl tumors. Perfusion in Veh-ctrl- or DC101-treated tumors was also limited to the periphery of the tumor (Figs. 5a, b). The aggregate data for DC101 treatment indicate that specifically targeting tumor vasculature (via VEGFR-2) in these primary pancreatic tumors has a minor effect in one tumor line, but that in general, the tumors in both groups behaved like Veh-ctrl tumors and continued expanding outwards from a central necrotic core.

Since Met-Gem and DC101 affected the tumor vasculature, and vascular growth and remodeling is a delicate balance of pro- and anti-angiogenic factors [33], selected molecular factors implicated in vascular reorganization were assayed (Additional File 2). Met-Gem reduced the levels of proangiogenic factors VEGF and PDGF-BB compared to Veh-ctrls, suggesting that the treatment has some antiangiogenic effect. VEGF is known to control endothelial cell growth, migration, and survival, but overexpression of VEGF typically causes abnormal, random, and disorganized vasculature [34, 35]. Since the levels of VEGF decreased with Met-Gem treatment, the tumor vasculature would be expected to become less haphazard and disorganized as seen here. The results reported here would appear consistent with the hypothesis originally put forth by Jain et al. [35] that metronomic therapy may induce vascular normalization. DC101 treatment increased the levels of both mouse and human VEGF, indicating that VEGFR-2 was successfully blocked, but somewhat surprisingly did not change vasculature patterns. Tumors are known to become resistant to antiangiogenic treatment by using alternative pathways to compensate for the inhibition of VEGFR-2 [36]. However, in this case, no change in P*l*GF, PDGF-BB, and SDF-1α was seen over the 3-week treatment period. It appears that the primary pancreatic tumors used in our study can survive VEGFR-2 inhibition without activating other pathways.

The differences in response to DC101 (a targeted drug) and Met-Gem (low-dose cytotoxic) provide some insight into how each affects tumor growth. The observations from tumors treated with Met-Gem are somewhat paradoxical since the tumors appear ‘healthier’—they had more functional blood vessels, less necrosis, and virtually no hypoxia—and yet the tumors did not proliferate, or at least proliferated slowly compared to Veh-ctrl- or DC101-treated tumors. Gemcitabine would initially cull proliferating endothelial and cancer cells to leave behind a tumor with mature blood vessels that are more functional and fewer cancer cells. The continuous delivery of gemcitabine subsequently appears to dampen the proliferative capacity of the cells. Since the drug is present more often during metronomic therapy, the cancer cells and endothelial cells may have no chance to recover as they do during chemotherapy breaks in conventional MTD therapy. The result is a tumor in stasis where cells are still viable, but less proliferative than normal. In contrast, targeting VEGFR-2 with DC101 in these primary tumors may not be effective because the VEGF pathway is not an important therapeutic target in pancreatic cancer [37], or because alternative pathways may be utilized. Similar results have also been reported in the clinic where targeted anti-VEGF treatment in pancreatic cancer was not successful, and failure of DC101 to Veh-ctrl tumors in our study mirrors these findings where inhibition of a single target was insufficient to achieve tumor control [38].

At present, the consequences of long-term Met-Gem treatment are unknown. The tumor may eventually be eradicated, or it could become refractory to Met-Gem and re-grow. However, it is tantalizing to speculate that if the treatment holds tumor growth in check indefinitely, Met-Gem treatment could be used as an additional therapeutic option where the goal is to attain a cytostatic state rather than to reduce tumor volume at all costs. Several intriguing implications arise from the effects of Met-Gem. Using non-invasive imaging technologies, as in our multi-modality study, may also be useful in determining therapeutic opportunities that arise from Met-Gem treatment. The homogenous perfusion due to improvements in vascular function may provide better access for a second drug to all cancer cells as was previously shown by our group [17] and could potentiate its cell-killing effects. DCE-MRI could be used to determine how sequential drug treatments could be used. The classic end point for drug activity is reduction of tumor volume [39]. However, new therapies that induce a cytostatic state [40] require other end points for the assessment of response and efficacy [41]. FDG-PET may be useful as a complementary measure of drug activity [42–44] because the technique is non-invasive, applicable to multiple scans, and measures a tumor’s metabolic activity which may change before tumor volumes are affected [45, 46]. This is exemplified by the FDG-PET scans in our study which show decreased cell viability before changes in tumor volume are observed after 3 days of treatment. There has only been a handful of studies of FDG-PET response with metronomic treatment of other drugs and other cancer models [47, 48], and its utility as an early surrogate response marker remains promising. We are the first to study FDG uptake with metronomic gemcitabine in pancreatic cancer and further studies will be required to confirm the predictive benefits of FDG-PET in this setting.

We have previously shown that Met-Gem has better efficacy at lower doses than conventional maximum tolerated doses [9]. In this study, we further confirmed that Met-Gem treatment is cytostatic and improves vasculature function transiently and that changes in the tumors’ viability can be detected before changes in their volume. DC101 treatment indicated that a treatment specific for VEGFR-2 may be less effective if the targeted pathway is no longer important in disease progression or if other pathways can compensate for its activity. However, a therapeutic strategy such as Met-Gem which provides the continuous presence of low-dose gemcitabine may control not only the proliferation of endothelial cells, but also cancer cells in a two-pronged attack. This approach appears to control cell growth well, as evidenced by the FDG-PET and Ki67 data, precisely because it is not targeted to a specific component of the disease.

There is clinical interest in combining metronomic therapies with dedicated anti-angiogenic agents as a therapeutic strategy for cancer treatments [49–53]. The therapeutic effects of combinations such as DC101 and metronomic vinblastine in neuroblastoma [8], DC101 with metronomic cisplatin or doxorubicin in breast cancer [54], and bevacizumab with metronomic irinotecan in colorectal cancer [55] have been examined in preclinical models. Phase I/II clinical studies have also been carried out in glioblastoma with bevacizumab and metronomic irinotecan [56, 57] and breast cancer with bevacizumab and metronomic cyclophosphamide [58]. The studies showed that the combination treatments are well tolerated, and in some cases produced stable disease. However, few conclusions can be made about the mechanisms of the combination therapies due to variation in study methods and small population patients. The consensus at the moment is that a better understanding of how metronomic therapies interact with anti-angiogenic therapies is needed and that more predictive biomarkers and imaging techniques are required to facilitate the sequencing (or monitoring effects) of the two treatments [49]. In our study, the data indicate that Met-Gem causes a transient period of improved perfusion and low hypoxia which could be advantageous for the delivery of a second drug, or radiation therapy, respectively. Met-Gem and DC101 were not combined in this study and so we are unable to comment on any potential synergies between the two treatments. However, our study also shows the utility of imaging technologies to assess changes in the physiology of the tumor to guide changes in initial treatment to take advantage of transient treatment opportunities in a combination or sequential setting.

## Electronic supplementary material

Below is the link to the electronic supplementary material.

Supplementary material 1 (PDF 585 kb)

Supplementary material 2 (PDF 53 kb)

## Abbreviations

FDG: ^18^F-Fluorodeoxyglucose
mSDF-1α: Mouse stromal cell-derived factor-1α
DCE-MRI: Dynamic contrast-enhanced magnetic resonance imaging
FDG-PET: ^18^F-fluorodeoxyglucose positron emission tomography
Gd-DTPA: Gadolinium diethylenetriamine penta-acetic acid
hP*l*GF: Human placental growth factor
hTSP-1: Human thrombospondin-1
Met-Gem: Metronomic gemcitabine
mPDGF-BB: Mouse platelet-derived growth factor-BB
MTD: Maximum tolerated dose
TUNEL: TdT-mediated nick end labeling
Veh-ctrl: Vehicle control
VEGF: Vascular endothelial growth factor
VEGFR-2: VEGF receptor 2
