# Supplementary material for: The differential effects of metronomic gemcitabine and antiangiogenic treatment in patient-derived xenografts of pancreatic cancer: treatment effects on metabolism, vascular function, cell proliferation, and tumor growth
Source: Angiogenesis. 2016 Mar 9;19:229–44. doi: 10.1007/s10456-016-9503-z (PMC4819514; doi:10.1007/s10456-016-9503-z)

Additional File 1. Metronomic gemcitabine-treated PaCa8 tumors have better perfusion, higher intratumoral microvessel density, and less hypoxia than DC101-treated tumors. Representative composite images of PaCa8 tumor sections on day 7 following initiation of treatment with vehicle (Veh-Ctrl; 0.9% saline), DC101 (800 µg, q3d), or metronomic gemcitabine (Met-Gem; 30 mg/kg, q3d). The presence of Hoechst 33362 (blue) indicates tissue perfusion. Intratumoral microvessels and hypoxia are detected by CD31 (red) and EF5 (green) immunofluorescence staining, respectively. Necrotic regions (pale green) are outlined in yellow.

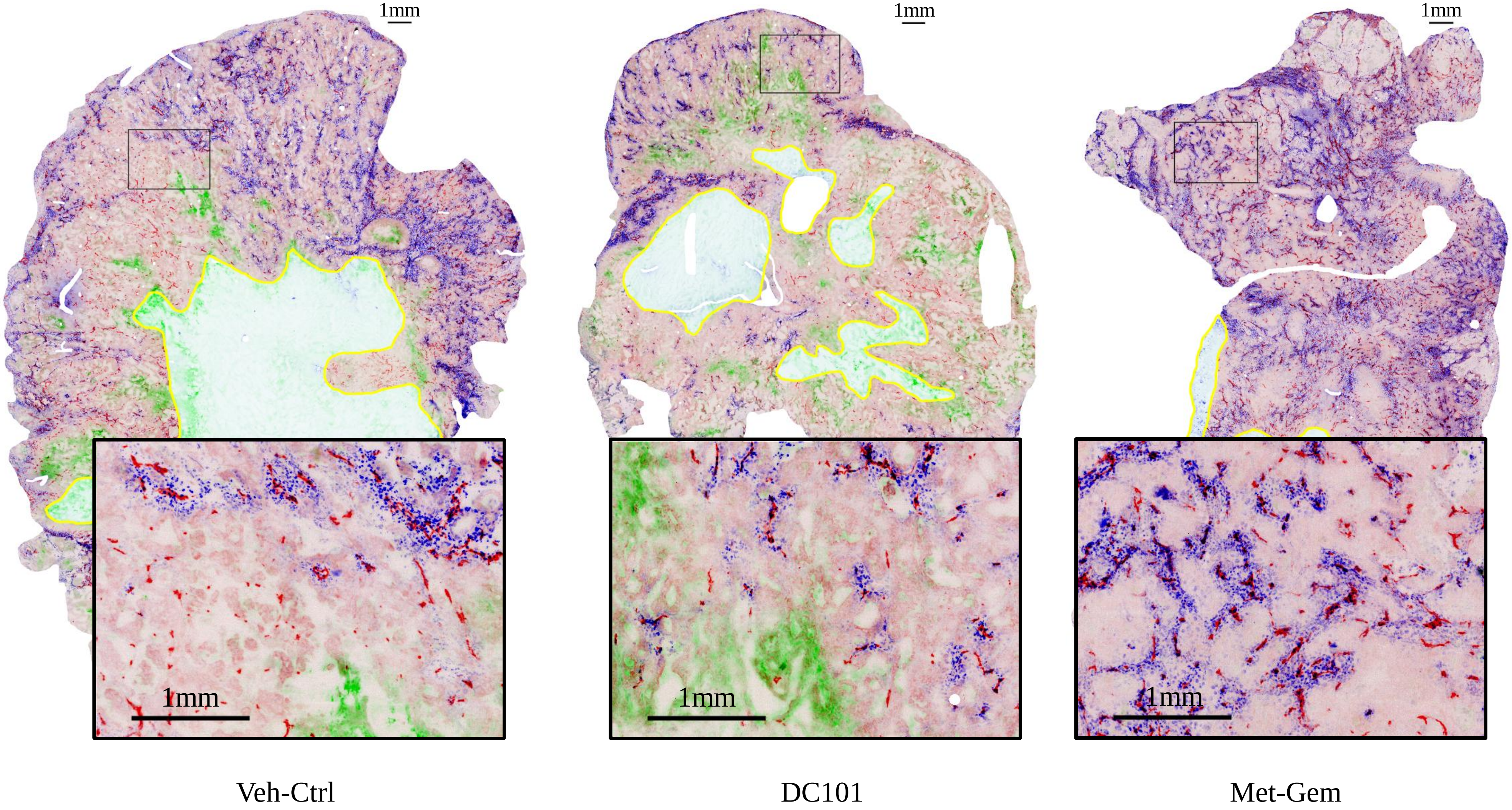

Supplement: Supplementary file 1 — Supplementary material 1 (PDF 585 kb) [file 10456_2016_9503_MOESM1_ESM.pdf]
