# Supplementary material for: The differential effects of metronomic gemcitabine and antiangiogenic treatment in patient-derived xenografts of pancreatic cancer: treatment effects on metabolism, vascular function, cell proliferation, and tumor growth
Source: Angiogenesis. 2016 Mar 9;19:229–44. doi: 10.1007/s10456-016-9503-z (PMC4819514; doi:10.1007/s10456-016-9503-z)
Supplement: Supplementary file 2 — Supplementary material 2 (PDF 53 kb) [file 10456_2016_9503_MOESM2_ESM.pdf]

Additional File 2. Protein levels of specific pro and antiangiogenic factors in the PaCa8 and PaCa13 tumors. hVEGF, human vascular endothelial growth factor; mVEGF, mouse vascular endothelial growth factor; hPLGF, human placental growth factor; mPDGF-BB, mouse platelet-derived growth factor; hSDF-1, human stromal-derived factor-1; hTSP-1, human thrombospondin-1. [a]P<0.05 *vs*. day 3 of corresponding treatment group. [b]P<0.05 *vs*. day 7 of corresponding treatment group. [c]P<0.05 *vs*. the vehicle control group at each time point. [d]P<0.05 *vs*. the DC101 group at each time point. [#]n ≤ 3 as some sample concentrations were too low to detect. Data are presented as mean±SEM.

PaCa8

pg/mg protein	**Day 3**			**Day 7**			**Day 21**		
	Veh-Ctrl	DC101	Met- Gem	Veh-Ctrl	DC101	Met- Gem	Veh-Ctrl	DC101	Met- Gem
hVEGF	205.3±30.9	215.0±25.9	77.4±9.4[c,d]	281.6±42.2	363.9±42.4[a]	56.5±9.7[c,d]	226.3±19.6	433.9±31.3[a,c]	29.5±7.8[c,d]
mVEGF	139.4±38.0	108.3±15.7	80.5±21.1	194.1±39.1	180.3±25.3	98.8±21.2	212.1±34.2	379.7±62.5[a,b,c]	129.3±26.1[d,#]
hPLGF	Concentrations too low to detect								
mPDGF-BB	129.8±18.9	124.9±19.9	66.4±11.0	171.1±32.8	171.1±32.8	40.4±6.3[a,c,d]	132.3±27.0	172.9±20.5	22.8±8.7[a,c,d]
mSDF-1α	88.7±26.6	94.7±15.7	87.7±16.2	90.4±13.5	94.7±17.2	167.1±16.9[c,d]	96.3±19.2	81.8±7.2[#]	143.4±60.5[#]
hTSP-1	Concentrations too low to detect								

PaCa13

	**Day 3**			**Day 7**			**Day 21**		
pg/mg protein	Veh-Ctrl	DC101	Met- Gem	Veh-Ctrl	DC101	Met- Gem	Veh-Ctrl	DC101	Met- Gem
hVEGF	134.6±30.8	235.6±37.8[c]	101.8±24.1[d]	147.4±25.6	345.4±83.3[c]	33.4±11.6[a,c,d]	207.1±44.9	316.0±67.5	45.6±7.7[a,c,d]
mVEGF	98.1±20.4	150.0±22.7	43.7±8.6[d]	103.3±12.2	241.0±17.0[c]	29.3±6.8[c,d]	125.1±30.0	223.7±48.4	40.8±8.7[d]
hPLGF	32.8±6.0	35.4±7.1	21.3±11.6	26.6±7.8	43.9±7.8	13.9[#]	46.9±16.6	17.0±3.5[b]	15.4±0.6[#]
mPDGF-BB	41.9±7.8	50.4±6.8	26.4±2.7[d]	57.0±10.4	54.9±8.8	22.9±4.3	50.6±11.9	37.4±5.1	31.0±4.8
mSDF-1α	142.7±27.7	151.5±28.7	149.7±24.2	152.8±21.9	134.6±18.3	137.7±30.9	223.0±50.1	206.1±49.6	257.5±53.3
hTSP-1 ($\times 10^5$)	10.3±1.5	12.0±0.7	10.0±1.6	12.3±2.4	8.8±1.1	4.1±1.0[a,c]	12.4±1.9	9.0±1.8	6.4±1.4[c]
